# Gabapentin Effects on PKC-ERK1/2 Signaling in the Spinal Cord of Rats with Formalin-Induced Visceral Inflammatory Pain

**DOI:** 10.1371/journal.pone.0141142

**Published:** 2015-10-29

**Authors:** Yan-bo Zhang, Zheng-dong Guo, Mei-yi Li, Peter Fong, Ji-guo Zhang, Can-wen Zhang, Ke-rui Gong, Ming-feng Yang, Jing-zhong Niu, Xun-ming Ji, Guo-wei Lv

**Affiliations:** 1 Department of Neurology, Affiliated Hospital of Taishan Medical University, Taian, China; 2 Department of Endocrinology, Affiliated Hospital of Taishan Medical University, Taian, China; 3 Department of Neurology, Shandong Taishan Chronic Disease Hospital, Taian, China; 4 Department of Neurology, University of California San Francisco, San Francisco, CA, United States of America; 5 Department of Pharmacology, College of Pharmacy, Taishan Medical University, Taian, China; 6 Department of Oral and Maxillofacial Surgery, University of California San Francisco, San Francisco, CA, United States of America; 7 Hypoxia Medical Institute, Xuanwu Hospital, Capital Medical University, Beijing, China; University of California, Los Angeles, UNITED STATES

## Abstract

Currently, the clinical management of visceral pain remains unsatisfactory for many patients suffering from this disease. While preliminary animal studies have suggested the effectiveness of gabapentin in successfully treating visceral pain, the mechanism underlying its analgesic effect remains unclear. Evidence from other studies has demonstrated the involvement of protein kinase C (PKC) and extracellular signal-regulated kinase1/2 (ERK1/2) in the pathogenesis of visceral inflammatory pain. In this study, we tested the hypothesis that gabapentin produces analgesia for visceral inflammatory pain through its inhibitory effect on the PKC-ERK1/2 signaling pathway. Intracolonic injections of formalin were performed in rats to produce colitis pain. Our results showed that visceral pain behaviors in these rats decreased after intraperitoneal injection of gabapentin. These behaviors were also reduced by intrathecal injections of the PKC inhibitor, H-7, and the ERK1/2 inhibitor, PD98059. Neuronal firing of wide dynamic range neurons in L6–S1 of the rat spinal cord dorsal horn were significantly increased after intracolonic injection of formalin. This increased firing rate was inhibited by intraperitoneal injection of gabapentin and both the individual and combined intrathecal application of H-7 and PD98059. Western blot analysis also revealed that PKC membrane translocation and ERK1/2 phosphorylation increased significantly following formalin injection, confirming the recruitment of PKC and ERK1/2 during visceral inflammatory pain. These effects were also significantly reduced by intraperitoneal injection of gabapentin. Therefore, we concluded that the analgesic effect of gabapentin on visceral inflammatory pain is mediated through suppression of PKC and ERK1/2 signaling pathways. Furthermore, we found that the PKC inhibitor, H-7, significantly diminished ERK1/2 phosphorylation levels, implicating the involvement of PKC and ERK1/2 in the same signaling pathway. Thus, our results suggest a novel mechanism of gabapentin-mediated analgesia for visceral inflammatory pain through a PKC-ERK1/2 signaling pathway that may be a future therapeutic target for the treatment of visceral inflammatory pain.

## Introduction

Visceral pain is characterized by inaccurate positioning, an imprecise nature of pain, and the frequent occurrence of referred pain. Approximately 10–40% of the general population suffers from visceral pain [1]. Recently, clinical examples of visceral pain, including inflammatory bowel disease and cancer-related visceral pain have become more prevalent, prompting greater interest in the mechanism and treatment of visceral pain. While the conduction of visceral pain is similar to that of somatic pain, both are categorized by different characteristics and clinical treatments. In contrast to the extensive literature of somatic pain, studies addressing the mechanism of visceral pain currently remain preliminary. Furthermore, the clinical therapeutic management of visceral pain remains less satisfactory than that of somatic pain, and studies investigating the mechanism of visceral pain with the aim of identifying new therapeutic targets have produced little success [1–5].

In 2002, the USA Food and Drug Administration approved gabapentin, a γ-aminobutyric acid derivative, for clinical use in the treatment of neuropathic pain. Since then, gabapentin has been extensively applied in the field of analgesia [6–9]. Preliminary data from animal experiments have confirmed the analgesic effects of gabapentin on visceral pain, but the mechanism of this effects remains unknown [10, 11]. In our previous study, we demonstrated the involvement of protein kinase C (PKC) and its subtypes, PKC γ and PKC ε, in the development and persistence of formalin-induced visceral inflammatory pain [9]. In addition, other work from Galan et al. and Sakurai et al. have elicited the role that extracellular signal-regulated kinase (ERK) signaling in the spinal cord and dorsal root ganglia contributes to the sensitization of visceral inflammatory pain [12, 13]. Thus, PKC and ERK1/2 signaling pathways may constitute novel molecular targets for the treatment of visceral inflammatory pain.

In a variety of pain models, including visceral pain, the persistence of chronic pain is known to occur through constitutive activation of N-methyl-D-aspartate (NMDA) receptors and calcium channels [14–16], which in turn, causes increased activation of PKC and ERK1/2 signaling, resulting in the chronic hyper-excitability of neurons involved in pain transmission [17]. In addition, the mechanism of gabapentin-mediated analgesia in treating pain has been shown to occur through the inhibition of NMDA receptors and calcium channels in the central nervous system [6–8, 18]. Therefore, we hypothesized that the gabapentin-induced analgesia in visceral inflammatory pain is mediated through an inhibitory effect on the PKC-ERK1/2 signaling. Previous reports from both our group and others have successfully modeled visceral inflammatory pain in the rodent by injecting formalin into the rat colonic submucosa [9, 19]. Therefore, we used the same model in this study to investigate the analgesic effect of gabapentin on visceral inflammatory pain and to test if this analgesic effect is mediated through a signaling pathway involving PKC and ERK1/2.

## Materials and Methods

### Experimental animals

A total of 240 rats (Male, Sprague-Dawley) weighing 200–250 g were provided by the Experimental Animal Center of Shandong at the University of Traditional Chinese Medicine in China (License number SCXK (Lu) 2005–0015). Rats were housed in a pathogen-free animal housing room at Taishan Medical University and fed with standard rat chow and tap water at 18–25°C. All protocols were approved by the Animal Care and Use Committee of Tai Shan Medical University in China and conducted in accordance with the Requirements for the Application of Pain in Conscious Animals, issued by the International Association for the Study of Pain. The number of animals used and the suffering of animals were kept to a minimum in all experiments. Rats were anesthetized with 4% isoflurane, and anesthesia was maintained with 2% isoflurane. A PE-10 polyethylene catheter (ID: 0.28 mm; OD: 0.61 mm; Becton Dickinson, USA) was inserted into the subarachnoid space through the atlanto-occipital membrane. After intubation, rats were administered with 2 mg of gentamicin via intramuscular injection once daily for 3 days and individually housed. Rats were monitored twice daily for 3 days, and those that could move freely, without any signs of infection, were used for the following experiments. Rats that experienced paralysis (about 30% of total animals) were euthanized with a lethal injection of sodium pentobarbital (Beijing Chemical Industry Group Co., Ltd., China). In this study, no animals died from infection or paralysis.

### Drug intervention

One hundred microliters of formalin (5%) were injected submucosally as described previously [9]. Thirty minutes before formalin injection in the colon, rats in the intrathecal catheter group were randomly divided into separate groups which received intrathecal administration of saline, 5% dimethyl sulfoxide (DMSO), H-7 (200 μg, dissolved in saline), PD98059 (5 μg, dissolved in 5% DMSO), or H-7 (200 μg) + PD98059 (5 μg). The drug injection volume was 20 μL followed by a flush with 10 μL saline. Rats in the intraperitoneal injection group were randomly injected with either saline or gabapentin (100 mg/Kg) 30 minutes before intracolonic injection of formalin.

### Behavioral observation

Rats were randomly assigned to 9 groups: intracolonic saline injection group (i.c.saline), intracolonic formalin injection group (i.c.F), intracolonic formalin injection + intrathecal saline injection group (i.c.F + i.t.saline), intracolonic formalin injection + intrathecal DMSO injection group (i.c.F + i.t.DMSO), intracolonic formalin injection + intraperitoneal saline injection group (i.c.F + i.p.saline), intracolonic formalin injection + intraperitoneal gabapentin injection group (i.c.F + i.p.GBP), intracolonic formalin injection + intrathecal H-7 injection group (i.c.F + i.t.H-7), intracolonic formalin injection + intrathecal PD98059 injection group (i.c.F + i.t. PD), and intracolonic formalin injection + intrathecal H-7 and PD98059 injection group (i.c.F+i.t. H-7 + i.t. PD). Each group contained 6 rats. A 1% Evans Blue solution was included in the formalin and saline solutions to visualize leakage. Rats with transmural injury were excluded from use in any experiments. After formalin injection, rats were placed in transparent cages, and pain behaviors were scored by an investigator who remained blinded to each rat’s treatment group. Pain behaviors included: L: abdominal licking and nibbling; B: stretching of the body, especially backward extension of the hind limbs; C: contraction of the flanks, sometimes evolving to a stretching attitude; and W: body contraction, curving of the back during standing, usually accompanied by abdominal cramps [19]. Results were calculated using a pain score formula revised from Miampamba et al, where S = 1L + 2B + 3C + 4W [19]. L, B, C and W represent the number of the corresponding behaviors over a period of time, and S represents the total pain score. Rat behavior was recorded for 120 consecutive minutes and scored every 15 minutes.

### Electrophysiological recordings

Rats were randomly divided into 7 groups: intracolonic formalin injection group (i.c.F), intracolonic formalin injection + intrathecal saline injection group (i.c.F + i.t.saline), intracolonic formalin injection + intraperitoneal saline injection group (i.c.F + i.p.saline), intracolonic formalin injection + intraperitoneal gabapentin injection group (i.c.F + i.p. GBP), intracolonic formalin injection + intrathecal H-7 injection group (i.c.F + i.t.H-7), intracolonic formalin injection + intrathecal PD98059 injection group (i.c.F + i.t. PD), and intracolonic formalin injection + intrathecal H-7 and PD98059 injection group (i.c.F + i.t. H-7 + i.t. PD). Each group contained 6 rats. Drugs that were administered intrathecally and intraperitoneally were given 30 minutes before intracolonic injections. Electrophysiological recordings in spinal dorsal horn neurons were performed in accordance with our previously described methods [9]. Briefly, extracellular recordings were conducted in the L6—S1 dorsal horn with a tungsten electrode (3–5 MΩ, gifts from Dr. William D. Willis). Wide dynamic range (WDR) neurons were identified by their various responses to colorectal expansionary stimulus. Neuronal signals were recorded and analyzed with a PowerLab system (AD Instrument Limited, Australia). Baseline neuronal firing rate in WDR neurons was defined as the number of action potentials recorded within the 15 minutes preceding formalin injection. Subsequent firing rates of these WDR neurons within the 15 minutes following formalin injection were measured and compared with this baseline. Throughout the procedure, rat core body temperatures were monitored with a rectal probe and maintained at 37°C with a servo-controlled heating blanket.

### PKC translocation to membrane

Rats were randomly assigned to 6 groups: intracolonic injection of saline (i.c.saline), intracolonic injection of formalin (i.c.F), intracolonic injection of formalin with intraperitoneal injection of saline group (i.c.F + i.p.saline), intracolonic injection of formalin with intraperitoneal injection of gabapentin (i.c.F + i.p.GBP), intracolonic injection of formalin with intrathecal injection of H-7 group (i.c.F + i.t.H-7) and intracolonic injection of formalin with intrathecal injection of PD98059 group (i.c.F + i.t.PD). Each group contained 6 rats. At 30, 60, and 120 minutes after formalin injection, laminectomy was performed to expose the spinal cord; and L6—S1 were quickly dissected and flash-frozen with liquid nitrogen. After cytoplasmic proteins and membrane proteins were extracted, PKC translocation to the membrane was detected in accordance with our previously described methods [9]. Protein samples were separated on a 10% gel, transferred to nitrocellulose membrane (Schleicher and Schell, USA), and incubated with PKC primary antibody (1:1000, sc-10800, Santa Cruz Biotechnology, Inc., USA), actin (1:1000, sc-47778, Santa Cruz Biotechnology, Inc., USA), and secondary antibody (1:5000, sc-2004 and sc-2005, Santa Cruz Biotechnology, Inc., USA). Membranes were then incubated in Supersignal West chemiluminescent reagents (Pierce, USA) to obtain a signal for exposure to radiographic film (Kodak, USA). GelDoc gel image analysis system (Bio-rad, USA) was used to perform western blot image scanning and optical density analysis. PKC membrane translocation was expressed as the ratio of PKC content in the cell membrane to total protein content (membrane protein + cytoplasmic protein).

### ERK1/2 phosphorylation

Rats were randomly assigned to 6 groups: normal control group (Normal), colonic saline injection group (i.c.saline), intracolonic formalin injection group (i.c.F), intracolonic formalin injection + intrathecal PD98059 injection group (i.c.F + i.t.PD), colonic formalin + intrathecal H-7 injection group (i.c.F + i.t.H-7), and intracolonic formalin injection + intraperitoneal gabapentin injection group (i.c.F + i.p.GBP). L6—S1 spinal cord segments were obtained at 30, 60 and 120 minutes following injection. For each time point, 6 rats were used for each group. Protein extracts were separated on 4–12% gradient gel, and ERK1/2 phosphorylation (1:1000, sc-135900 for ERK and sc-101760 for p-ERK, Santa Cruz Biotechnology, Inc., USA) was measured according to our previously described methods [20]. After detection of ERK1/2 protein levels, the nitrocellulose membrane was treated with a second western blot hybridization to measure levels of phosphorylated ERK1/2 (p-ERK1/2) protein. Relative ERK1/2 phosphorylation levels were expressed as the ratio of p-ERK1/2 to total ERK1/2 protein.

### Data analysis and statistics

Statistical analyses were performed with Sigmaplot (V12.5, Systat Software Inc, San Jose, CA). One-way ANOVA with Bonferroni’s post -hoc test was used to analyze the Western blot data. Two-way repeated measures ANOVA with Dunnett’s post-hoc test was used to test significance of differences between groups for behavior and electrophysiology data. p -values less than 0.05 were considered significant. Data are reported as mean ± SEM.

## Results

### Gabapentin, H-7, and PD98059 suppressed pain behavioral responses of rats with formalin-induced visceral inflammatory pain

**Fig 1:**
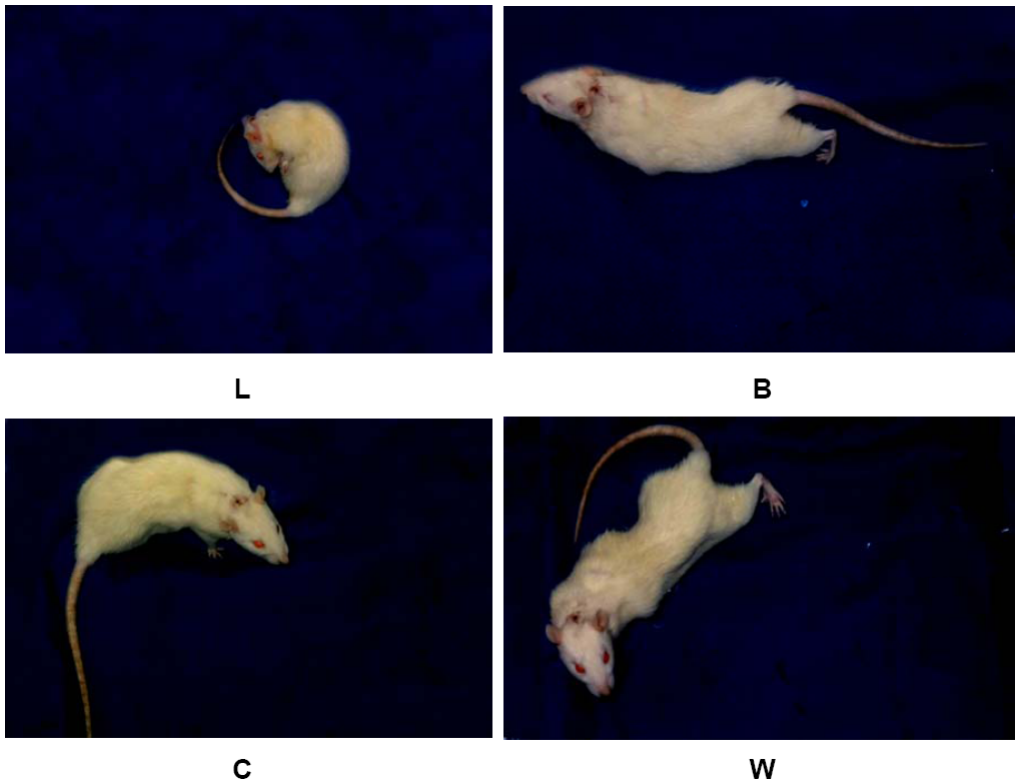
The four characteristic pain behaviors following intracolonic (i.c.) injection of formalin. L: abdominal licking and nibbling, B: stretching of the body, especially backward extension of the hind limbs, C: contraction of the flanks, sometimes evolving to a stretching attitude, W: body contraction, curving of the back during standing, usually accompanied by abdominal cramps.

**Fig 2:**
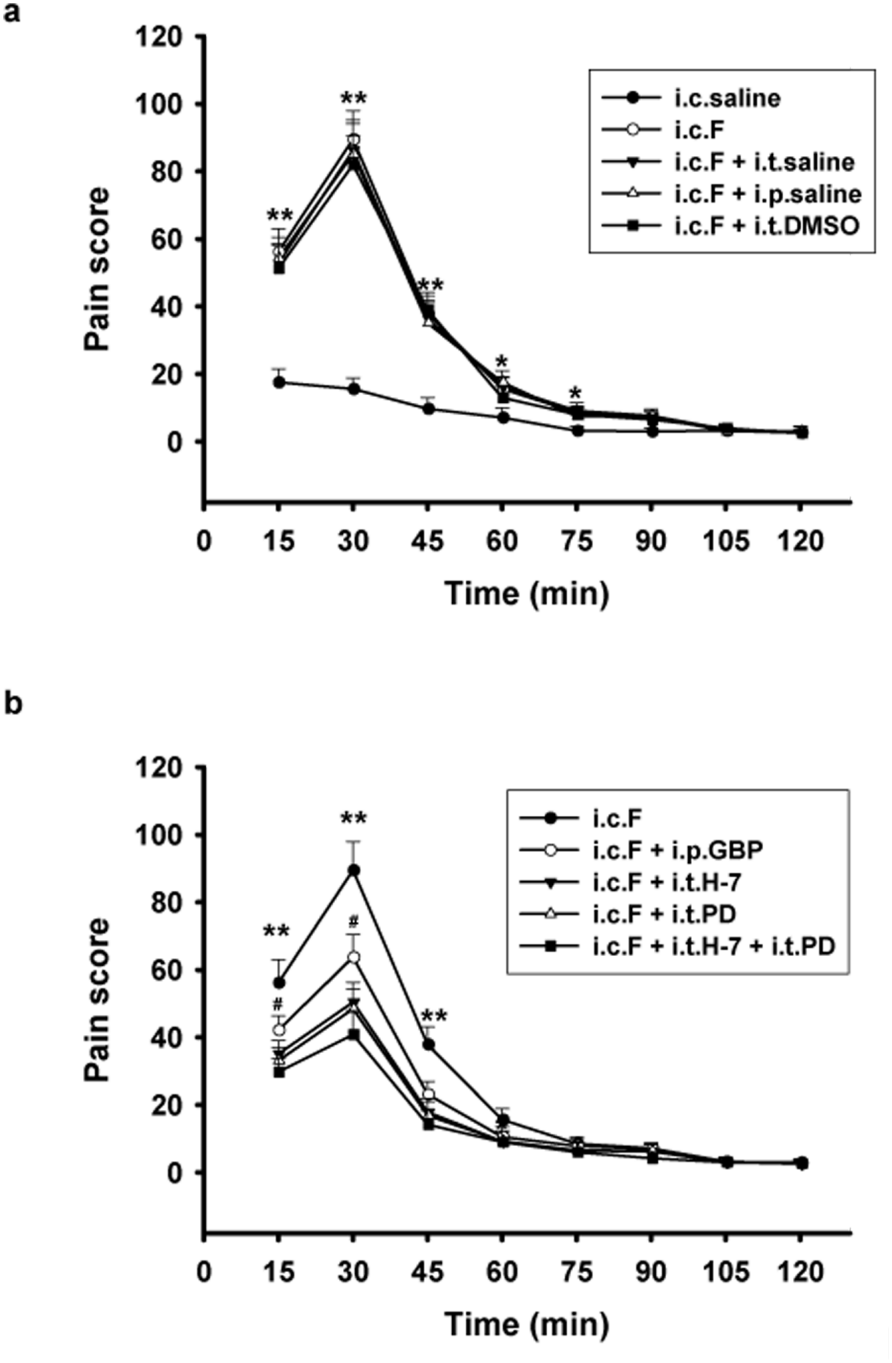
Administration of gabapentin (GBP), H-7, and PD98059 (PD) suppressed visceral pain behaviors induced by intracolonic injection of formalin. (a) Intracolonic injection of formalin induced observable visceral pain behaviors that lasted for at least 75 min. Neither intraperitoneal (i.p.) nor intrathecal (i.t.) injection of vehicle control (saline or DMSO) had a significant effect on pain behaviors. * p < 0.05, ** p < 0.01, compared with control group that received intracolonic injection of saline (i.c.saline). (b) I.p. injection of GBP significantly reduced visceral pain, as illustrated by the reduced pain scores following formalin injection. I.t. administration of H-7 and PD, both individually and combined, also significantly reduced visceral pain behaviors and exhibited a stronger effect than that of GBP. ** p < 0.01, i.c.F group vs. all other groups; # p < 0.05, i.c.F + i.p.GBP vs. i.c.F + i.t.H-7, or i.t.PD or i.t.H-7 + i.t.PD. n = 6 for each group.

Intracolonic injection of saline induced mild pain behaviors (L reaction, Fig 1) that developed within the first 15 minutes following injection. These behaviors gradually decreased and subsided at 60 minutes following injection (Fig 2a). However, injection of formalin into the colonic submucosa produced prominent pain behaviors in rats immediately after they regained consciousness. Within the first 60 minutes following formalin injection, rat pain behaviors primarily consisted of W, C, and B type behaviors (Fig 1). During the second 60 minutes following injection, the majority of pain behaviors were L type. Following formalin injection, pain scores were 56.23 ± 6.80 during the first 15 minutes and 89.52 ± 8.45 during the second 15 minutes, reaching a maximum at 30 minutes post injection. During this time period, pain scores from formalin-injected rats were significantly higher than those from saline-injected control rats (p = 0.000, df = 1, F = 2113.786, Fig 2a). After 30 minutes, pain scores remained higher among formalin-injected rats, but scores in both groups began to diminish until 75 minutes post-injection (7.20 ± 1.55 vs. 1.51 ± 0.21, p < 0.05). After 75 minutes following injection, both formalin and saline-injected rats continued to demonstrate intermittent pain behaviors with no statistically significant difference between the two groups (Fig 2a).

Neither intrathecal administration of vehicle control (saline or DMSO) nor intraperitoneal injection of saline affected formalin-induced visceral pain behaviors in rats (p > 0.05; Fig 2a). However, intraperitoneal injection of gabapentin inhibited pain behaviors within the first 45 minutes following formalin injection (p = 0.000, df = 1, F = 1422.150, compared with formalin injection group, Fig 2b). At 30 minutes following formalin injection, gabapentin decreased pain behavior scores from 91.52 ± 23.17 to 63.57 ± 19.87 (p = 0.0052). At 60 minutes following formalin injection, pain scores from gabapentin-treated rats no longer showed any statistical difference from those of untreated rats in the formalin group. To test the hypothesis that PKC and ERK are involved in the mechanism of visceral pain, the PKC inhibitor, H-7 and the EKR inhibitor, PD98059 were administered intrathecally in rats following formalin injection. Administration of H-7 and PD98059, both alone and combined, significantly inhibited behavioral pain responses and decreased pain scores in rats with visceral inflammatory pain (p < 0.01, compared with formalin injection group, Fig 2b). The analgesic effects of H-7 and PD98059, both alone and combined, were greater than that of gabapentin in the first 30 minutes following formalin injection, as shown in Fig 2b (p < 0.05, i.c.F + i.p.GBP vs. i.c.F + i.t.H-7, PD, or H-7 + PD, Fig 2b).

### Changes in the excitability of wide dynamic range neurons induced by formalin and the effects of gabapentin, H-7, PD98059, and H-7 + PD98059

**Fig 3:**
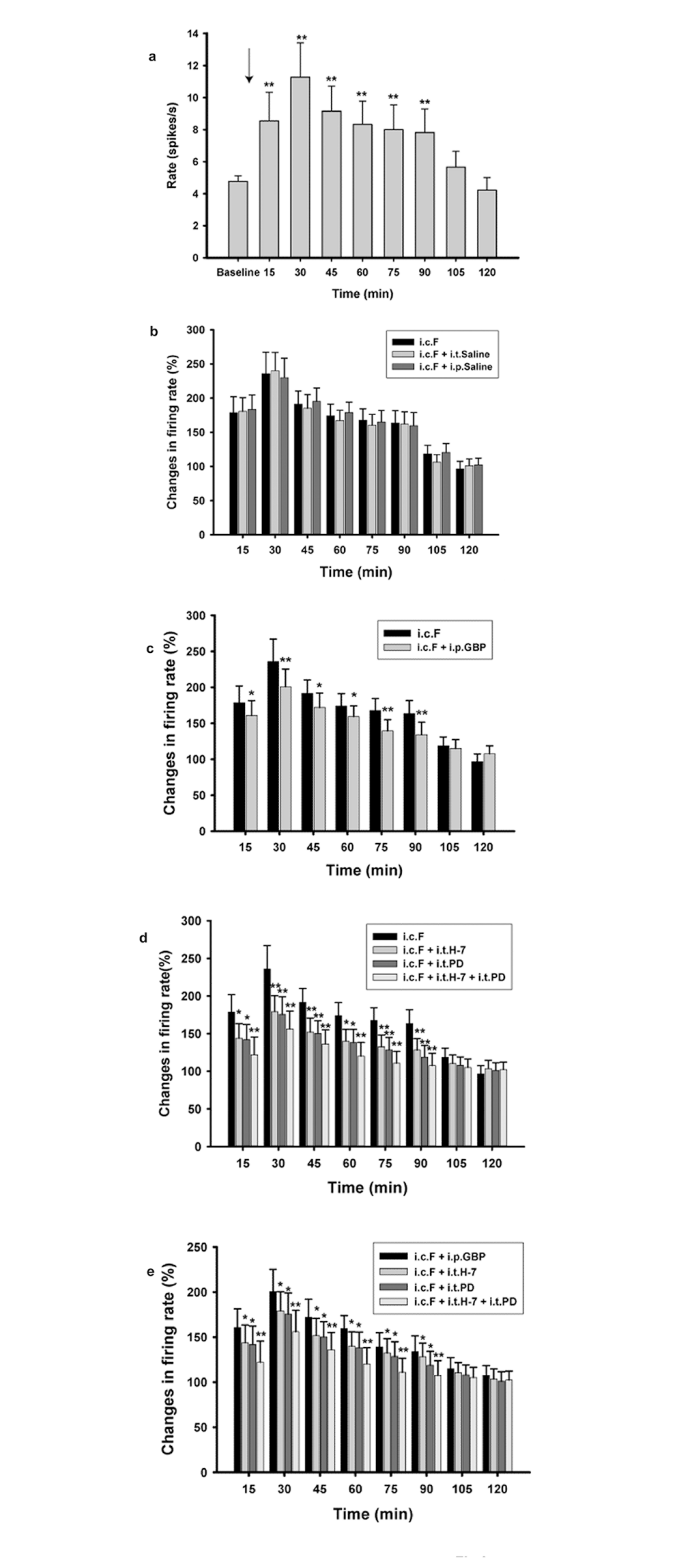
Administration of gabapentin (GBP), H-7, and PD98059 (PD) inhibited the increased firing of wide dynamic range neurons following intracolonic injection of formalin. (a) Firing rate of wide dynamic range (WDR) neurons in the dorsal horn increased following formalin injection, reaching a maximum at 30 minutes and remaining higher than baseline firing rate until 90 minutes post injection. Arrow indicates the time of formalin injection. ** p < 0.01, compared with baseline. (b) Neither intrathecal (i.t.) nor intraperitoneal (i.p.) administration of saline significantly affected the firing rate of WDR neurons following intracolonic formalin injection (i.c.F). (c) I.p. injection of GBP significantly inhibited the firing rate of WDR neurons following formalin injection. * p < 0.05, ** p < 0.01, compared with firing rates in rats receiving only formalin injection. (d) I.t. administration of H-7, PD, and H-7 with PD combined significantly inhibited the firing rate of WDR neurons following formalin injection. * p < 0.05, ** p < 0.01, compared with the firing rates in rats receiving formalin injection only. (e) I.t. administration of H-7, PD, and H-7 and PD combined produced significantly greater inhibitory effects than i.p. injection of GBP did on the elevated firing rates of WDR neurons following formalin injection. * p < 0.05, ** p < 0.01, compared with the firing rates in rats receiving i.p. injections of GBP. n = 6 for each group.

In total, 42 wide dynamic range neurons from 42 rats were recorded for all electrophysiology experiments. All recorded neurons were localized to 100–500 μm below the dorsal surface of the spinal cord. After formalin injection, neuronal firing increased and reached a maximum at 30 minutes with a mean firing frequency (11.27 ± 2.14Hz) that was twice that of the baseline firing frequency (4.78 ± 0.34Hz) (p *=* 0.0029, Fig 3a). After 30 minutes, neuronal firing rate decreased but remained significantly greater than baseline firing frequency. After 90 minutes, neuronal firing rate continued to decrease but was not significantly different from baseline firing rate (Fig 3a). Neither intrathecal nor intraperitoneal injection of saline affected neuronal firing rate in the spinal cord of rats with visceral inflammatory pain (p = 0.895, repeated measures two way ANOVA; Fig 3b). However, in the 90 minutes following formalin injection, gabapentin (i.p) significantly reduced neuronal firing frequency (p < 0.05 or p < 0.01, n = 6, Fig 3c). Similarly, intrathecal injection of H-7, PD98059, and H-7 + PD98059 also significantly inhibited the increased firing rate induced by formalin injection (p < 0.05 or p < 0.01, n = 6, Fig 3d, repeated measures two way ANOVA). At 30 minutes following formalin injection, H-7 and PD98059, alone, reduced the neuronal firing rate from 230.5 ± 47.2% to 181.6 ± 24.7% and 175.3 ± 28.9%, respectively, while H-7 and PD98059, combined, reduced it to 158.1 ± 31.2% (p = 0.0084, compared with i.c.F, n = 6, Fig 3d). Although the firing rate was further reduced from the combined application of H-7 and PD98059, statistical analysis did not reveal a significant difference between this effect and that from the application of either H-7 or PD98059 alone (Fig 3d). Thus, gabapentin, H-7, and PD98059 all inhibited the formalin-induced increase in firing rate of dorsal spinal cord neurons, with gabapentin producing the weakest inhibitory effect (Fig 3e).

### Gabapentin suppresses membrane translocation of PKC in the spinal cord of rats with visceral inflammatory pain

**Fig 4:**
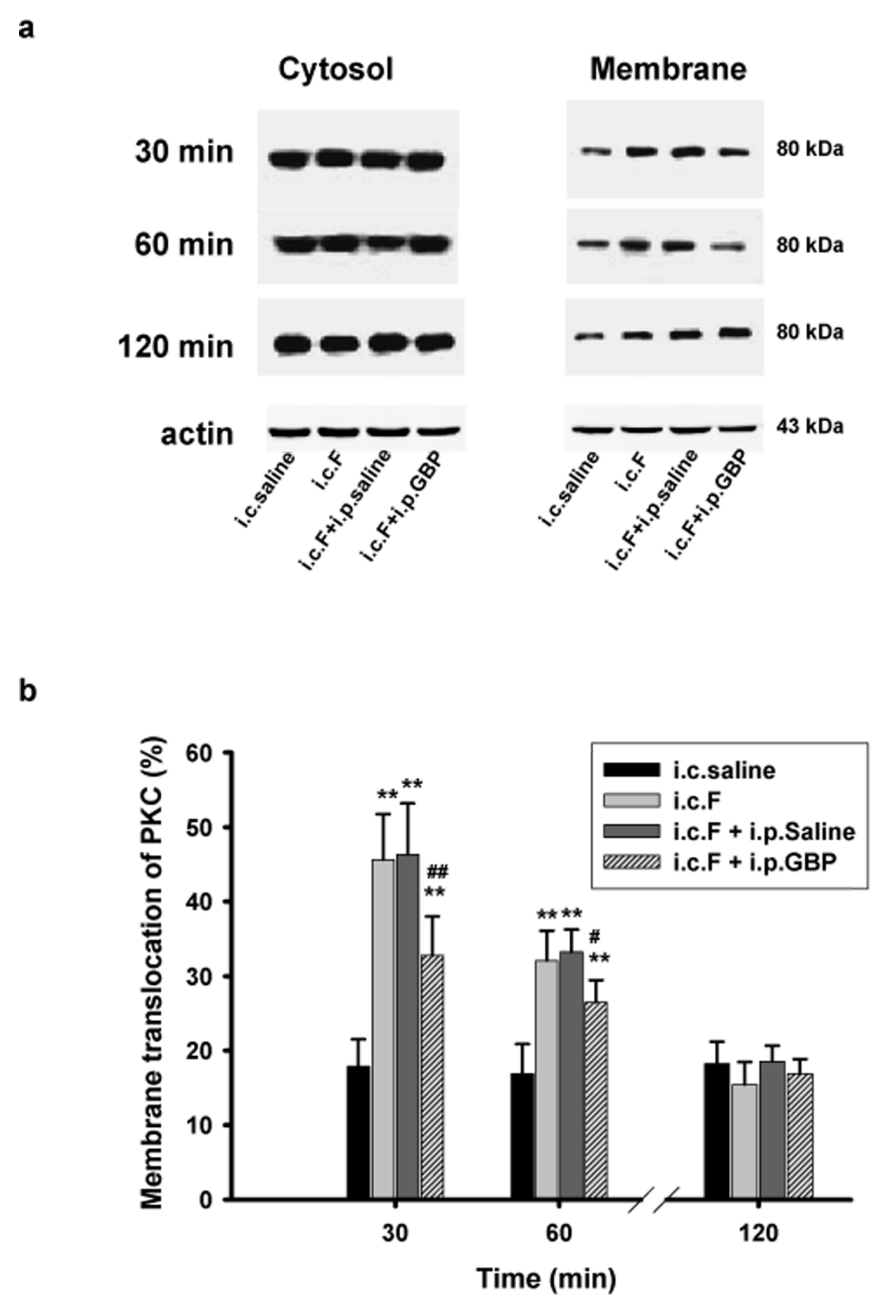
Intraperitoneal injection of Gabapentin (GBP) inhibited membrane translocation of PKC in the spinal cord of rats with visceral inflammatory pain. (a) Immunoblots showing membrane translocation of PKC from each treatment group at 30, 60 and 120 minutes following intracolonic injection. Left panels: immunoblots from cytosol fraction. Right panels: immunoblots from membrane fraction. (b) Statistical analyses showing that i.p. GBP inhibited membrane translocation of PKC at 30 and 60 minutes following intracolonic injection of formaline. Membrane translocation was expressed as the ratio (percentage) of membrane protein to total protein (membrane + particulate). ** p < 0.01, vs. saline control group, # p < 0.05, ## p < 0.01, vs. formalin group.

Behavioral tests revealed that pain was most prominent at 30 minutes following formalin injection and no longer statistically different between groups at 60 minutes following injection. Therefore, rat spinal cords were dissected at 30, 60, and 120 minutes after intracolonic injection and analyzed for membrane translocation of PKC. Rats receiving intracolonic formalin injection demonstrated significantly higher levels of PKC membrane translocation at 30 minutes (45.7 ± 6.1%) and 60 minutes (32.1 ± 4.0%) post injection than control rats that received intracolonic saline injection (17.9 ± 3.7%) (p = 0.008; Fig 4a and 4b). At 30 and 60 minutes post formalin injection, gabapentin significantly inhibited the membrane translocation of PKC to 31.9 ± 9.8% and 27.1 ± 4.1%, respectively (p < 0.01, or p < 0.05, n = 6, vs. i.c.F or i.c.F + i.p.Saline. one way ANOVA). At 120 minutes post injection, no significant differences in PKC membrane translocation were detected among any of the different groups (p > 0.05; Fig 4a and 4b). Interestingly, while the PKC inhibitor, H-7, inhibited pain behavioral responses and the increased neuronal firing associated with visceral pain, it did not reduce PKC membrane translocation at any of the three time points (S1 Fig). This result can most likely be attributed to the fact that H-7 functions to inhibit PKC by blocking ATP-binding sites located on the C3–4 domains of PKC, while PKC membrane translocation is determined by the C1–2 domains [21–23]. Thus, our results are consistent with the mechanism of H-7 mediated PKC inhibition, since it should not interfere with PKC membrane translocation. Similarly, the ERK inhibitor, PD98059, did not affect PKC membrane translocation either (S1 Fig), as we expected.

### Gabapentin, H-7, and PD98059 decreased ERK1/2 phosphorylation levels in the spinal cord of rats with visceral inflammatory pain

**Fig 5:**
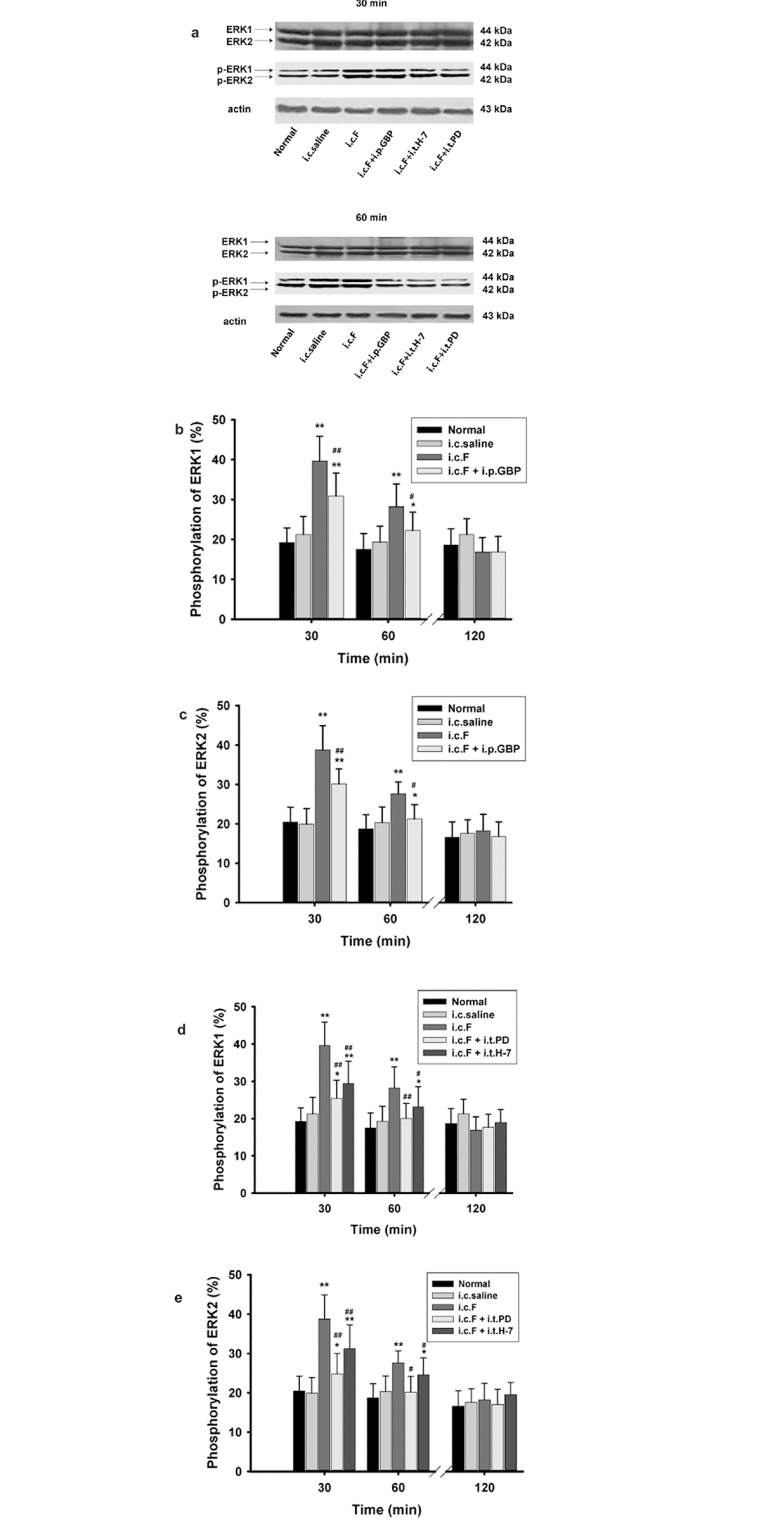
Gabapentin (GBP), H-7, and PD9809 (PD) reduced ERK1/2 phosphorylation in the spinal cord of rats with visceral inflammatory pain. (a) Immunoblots showing ERK1/2 phosphorylation from each treatment group at 30 minutes (upper panels) and 60 minutes (lower panels) following intracolonic injection. Statistical analyses also showing that GBP inhibited the increased phosphorylation of ERK1 (b) and ERK2 (c) at 30 and 60 minutes. following intracolonic formalin injection. I.t administration of PD and H-7 also inhibited the increased phosphorylation of ERK1 (d) and ERK2 (e) at 30 and 60 minutes following intracolonic formalin injection. ERK phosphorylation was expressed as the ratio (percentage) of p-ERK1/2 to total ERK1/2. * p < 0.05, ** p < 0.01, vs. normal control group or saline group; # p < 0.05, ## p < 0.01, vs. formalin injection group.

In conjunction with our investigation of PKC membrane translocation, rat spinal cords were obtained at 30, 60, and 120 minutes following intracolonic injection and analyzed for ERK1 and ERK2 phosphorylation levels. At 30 minutes following injection, ERK1 and ERK2 phosphorylation was significantly higher in formalin-injected rats than in either normal control rats or saline-injected control rats (p < 0.05, p < 0.01; Fig 5a–5c). At 30 and 60 minutes following formalin injection, Gabapentin treatment markedly reduced ERK1 and ERK2 phosphorylation. Similarly, treatment with PD98059 significantly diminished ERK1 and ERK2 phosphorylation 30 minutes after formalin injection (p = 0.009; Fig 5d and 5e), while treatment with H-7 produced a similar effect at 30 and 60 minutes post-injection (p < 0.05, p < 0.01; Fig 5d and 5e). After 120 minutes, no significant differences in ERK1 or ERK2 phosphorylation levels were observed between formalin-injected rats and either normal control rats or saline-injected control rats (p < 0.05, p < 0.01; Fig 5b and 5c).

## Discussion

In this study, we demonstrated that visceral inflammatory pain induced by intracolonic injection of formalin is significantly inhibited by gabapentin. Western blot analysis showed that the activation of PKC and ERK1/2 in visceral inflammatory pain is also blocked by gabapentin, suggesting that the analgesic effect of gabapentin is mediated through suppression of PKC and ERK1/2 signaling.

Colonic submucosal injection of formalin is commonly used as a reliable model of visceral inflammatory pain [9, 19]. In the present study, pain behaviors resulting from intracolonic submucosal injection of formalin reached a maximum at 30 minutes following injection and gradually decreased, but these behaviors remained prominent until 75 minutes following injection. The time course of these effects are consistent with previous reports [19], indicating the validity of this model in producing visceral inflammatory pain. In addition, pain scores were also consistent with changes in the firing rate of WDR neurons in the spinal cord, which similarly peaked at 30 minutes post injection and gradually decreased until there was no significant difference with baseline firing at 90 minutes post injection (Fig 3). Furthermore, central sensitization, which involves the hyperactivation of WDR neurons in the spinal cord and has been widely documented in somatic pain [24], might also play an important role in the development of visceral pain. Previous reports have shown that wide dynamic range neurons in the spinal cord are sensitized in a PKC-dependent manner [9, 19]. Results from the current study demonstrate that the PKC inhibitor, H-7, as well as the ERK1/2 inhibitor, PD98059, are sufficient in inhibiting pain behavioral responses, as well as in suppressing the increased neuronal firing of WDR neurons and the elevated ERK1/2 phosphorylation associated with visceral inflammatory pain. Thus, both PKC and ERK1/2 signaling critically contribute to the induction and maintenance of visceral inflammatory pain.

In a previous study, we confirmed that PKC and its subtypes, PKC γ and PKC ε, participate in the development and persistence of formalin-induced visceral inflammatory pain in the rat spinal cord [9]. Our current report further reveals the involvement of the ERK1/2 signaling pathway in visceral pain that is consistent with studies by Galan et al. and Sakurai et al., which also report the role of ERK in the spinal cord and spinal ganglia in maintaining the sensitization of pain in visceral inflammatory pain [12, 13]. Our data show that ERK activation is blocked by both an ERK1/2 inhibitor and a PKC inhibitor (Fig 5c and 5d), indicating that both PKC and ERK1/2 are involved in the signaling pathway of visceral pain [25]. In addition, the analgesic effect of the combined application of these inhibitors was not significantly different from that of the application of either inhibitor alone. Therefore, these data support the implication that PKC and ERK1/2 are involved in the same signaling pathway of visceral pain, rather than in parallel pathways [26–31].

Although gabapentin has been extensively applied clinically to treat neuropathic pain [32–35], studies demonstrating its analgesic effect in treating visceral pain are limited. While some reports have confirmed the use of gabapentin in effectively treating visceral pain [36–38], the mechanism of this effect remains largely unknown. Through Western blot analysis, our data show that gabapentin significantly diminishes the membrane translocation of PKC and the phosphorylation levels of ERK1/2, suggesting that the analgesic effect of gabapentin is mediated through the suppression of PKC and ERK1/2 signaling pathways. In addition, numerous studies have identified gabapentin as a NMDA receptor blocker and a voltage-gated calcium channel blocker [6–8]. NMDA and calcium receptor activation in the peripheral and central nervous systems have been shown to be involved in the development and maintenance of various pain states, including neuropathic pain and visceral pain [14, 15, 39]. Thus, one possible mechanism for the analgesic effect of gabapentin is the blockade of the increased intracellular calcium and diacylglycerol that is required for the activation of PKC, thereby inhibiting the positive feedback loop of PKC activation [21] and the PKC-mediated ERK activation present during pain [40]. Therefore, the inhibitory effect of gabapentin on ERK phosphorylation in our current study might arise from either direct effects on ERK signaling or indirect effects mediated through PKC signaling. In addition to this proposed mechanism, gabapentin may also ameliorate visceral pain through other mechanisms. Many inflammatory cytokines, including TNF alpha, IL-1beta, and inflammation-related enzymes such as cyclooxygenase-2 are known to be activated in visceral inflammation [41, 42] and inhibited by gabapentin [43]. Therefore, these targets provide an additional interesting source of investigation for future studies.

This study demonstrated the significant analgesic effects of gabapentin, H-7, PD98059, and H-7+PD98059 in the treatment of visceral pain, with H-7 and PD98059 producing greater effects than that of gabapentin. Our results indicate the robust analgesia produced by PKC and ERK pathway-specific inhibitors in diminishing visceral inflammatory pain, providing evidence for signaling molecules within these pathways to be novel molecular targets for the treatment of visceral pain. Interestingly, co-application of H-7 and PD98059 did not reveal a significantly greater analgesic effect than the application of either treatment alone, and treatment with H-7 in formalin-injected rats markedly reduced ERK1/2 activation, suggesting that PKC and ERK are upstream/downstream of a common signaling pathway of visceral pain.

In summary, our study confirmed the analgesic effect of gabapentin, a treatment for neuropathic pain, in reducing formalin-induced visceral pain. In addition, we provided evidence for the critical role of PKC and ERK1/2 signaling in the pathogenesis of visceral inflammatory pain. Our results suggest a mechanism of gabapentin-mediated analgesia in visceral pain that involves the suppression of PKC and ERK1/2 signaling. Furthermore, these data implicate that PKC and ERK1/2 signaling contribute to a common pathway of visceral pain that may include novel therapeutic targets for the future research and treatment of visceral pain.

## Supporting Information

S1 Fig Intrathecal administration of H-7 and PD98059 (PD) do not inhibit the membrane translocation of PKC caused by intracolonic injection of formalin. (a) Immunoblots showing PKC membrane translocation from each treatment group at 30, 60 and 120 minutes following intracolonic injection. (b) Statistic analysis showing no significant difference in PKC membrane translocation among rats pretreated with H-7, PD, or H-7+ PD before intracolonic formalin injection and rats pretreated with saline control. ** p < 0.01, saline group vs the other three groups.
(TIF)
