# Supplementary figures and images for: Gabapentin Effects on PKC-ERK1/2 Signaling in the Spinal Cord of Rats with Formalin-Induced Visceral Inflammatory Pain
Source: PLoS One. 2015 Oct 29;10(10):e0141142. doi: 10.1371/journal.pone.0141142 (PMC4626203; doi:10.1371/journal.pone.0141142)

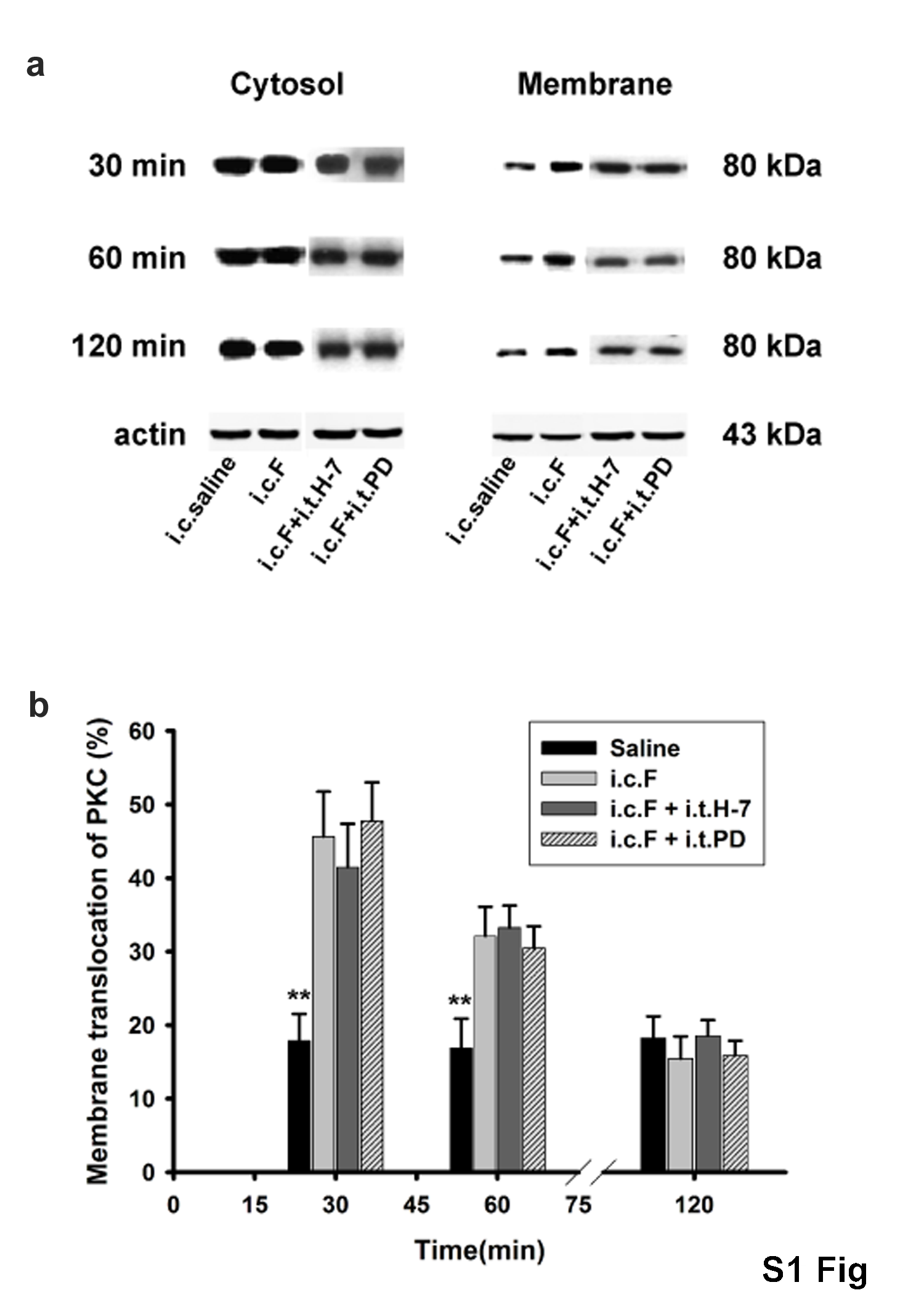

Supplement: S1 Fig — (a) Immunoblots showing PKC membrane translocation from each treatment group at 30, 60 and 120 minutes following intracolonic injection. (b) Statistic analysis showing no significant difference in PKC membrane translocation among rats pretreated with H-7, PD, or H-7+ PD before intracolonic formalin injection and rats pretreated with saline control. ** p < 0.01, saline group vs the other three groups. (TIF) [file pone.0141142.s001.tif]
